# Insight Into Metabolic Versatility of an Aromatic Compounds-Degrading *Arthrobacter* sp. YC-RL1

**DOI:** 10.3389/fmicb.2018.02438

**Published:** 2018-10-11

**Authors:** Lei Ren, Yang Jia, Rui Zhang, Zhong Lin, Zhen Zhen, Hanqiao Hu, Yanchun Yan

**Affiliations:** ^1^Agricultural College, Guangdong Ocean University, Zhanjiang, China; ^2^Graduate School of Chinese Academy of Agricultural Sciences, Beijing, China; ^3^Shenzhen Research Institute of Guangdong Ocean University, Shenzhen, China; ^4^Faculty of Chemistry and Environmental Science, Guangdong Ocean University, Zhanjiang, China

**Keywords:** aromatic compounds, biodegradation, metabolic versatility, dioxygenase, biphenyl

## Abstract

The genus *Arthrobacter* is ubiquitously distributed in different natural environments. Many xenobiotic-degrading *Arthrobacter* strains have been isolated and described; however, few have been systematically characterized with regard to multiple interrelated metabolic pathways and the genes that encode them. In this study, the biodegradability of seven aromatic compounds by *Arthrobacter* sp. YC-RL1 was investigated. Strain YC-RL1 could efficiently degrade *p*-xylene (PX), naphthalene, phenanthrene, biphenyl, *p*-nitrophenol (PNP), and bisphenol A (BPA) under both separated and mixed conditions. Based on the detected metabolic intermediates, metabolic pathways of naphthalene, biphenyl, PNP, and BPA were proposed, which indicated that strain YC-RL1 harbors systematic metabolic pathways toward aromatic compounds. Further, genomic analysis uncovered part of genes involved in the proposed pathways. Both intradiol and extradiol ring-cleavage dioxygenase genes were identified in the genome of strain YC-RL1. Meanwhile, gene clusters predicted to encode the degradation of biphenyl (*bph*), para-substituted phenols (*npd*) and protocatechuate (*pca*) were identified, and *bphA1A2BCD* was proposed to be a novel biphenyl-degrading gene cluster. The complete metabolic pathway of biphenyl was deduced via intermediates and functional gene analysis (*bph* and *pca* gene clusters). One of the these genes encoding ring-cleavage dioxygenase in *bph* gene cluster, a predicted 2,3-dihydroxybiphenyl 1,2-dioxygenase (BphC) gene, was cloned and its activity was confirmed by heterologous expression. This work systematically illuminated the metabolic versatility of aromatic compounds in strain YC-RL1 via the combination of metabolites identification, genomics analysis and laboratory experiments. These results suggested that strain YC-RL1 might be a promising candidate for the bioremediation of aromatic compounds pollution sites.

## Introduction

Widespread environmental pollution and tightening environmental regulations have increased demand for environmental remediation and driven research on pollutant degradation. Biodegradation is recognized as a promising approach for eliminating pollutants from some environmental settings (Boxall et al., 2004; Singh, 2008). Whereas eukaryota mostly transform xenobiotics for detoxification or via fortuitous transformation by broad-spectrum enzymes, prokaryota typically metabolize them for assimilation as essential nutrients and energy (Copley, 2009; Fenner et al., 2013). Numerous bacteria have been isolated and characterized with respect to the molecular mechanisms underlying their metabolic potential for the degradation of environmental pollutants (Copley, 2009; Fenner et al., 2013). Despite early optimism, biodegradation has not been a panacea for environmental pollution. Many issues require further investigation before its full potential can be revealed. For example challenges remain regarding the degradation of pollutants present at low concentrations, present in extreme conditions (i.e., saline-alkali soil, deserts, etc.), present in mixtures with other compounds, and those that might be toxic or metabolized to toxic intermediates (Yagi et al., 2009; Laffin et al., 2010; Narancic et al., 2012).

As one of the most common structural units of organic compounds in nature, the benzene ring and its derivatives are extensively used in industrial production, including daily supplies, agro-products, energy products, etc. Although some structural properties of these compounds make them recalcitrant to degradation, many aromatic-degrading microbes have been isolated (Fuchs et al., 2011; Xu et al., 2015), with metabolic pathways and mechanisms reported for some (Chauhan et al., 2000; Zhang et al., 2012; Shah et al., 2014; Gran-Scheuch et al., 2017), but not all (Goncalves et al., 2006; Zhou et al., 2015). These works and hundreds of others have advanced our knowledge of aromatic compounds degradation. Some of these isolates could degrade several kinds of xenobiotics and have attracted much attention from researchers (**Supplementary Table S1**). This topic is particularly relevant to environmental remediation since pollutants rarely occur in isolation at contaminated sites, but are rather more likely to part of mixture of compounds. There is, therefore a pressing need to study the related mechanisms of metabolic versatility which is more relevant to the *in situ* situation.

The genus *Arthrobacter* was firstly isolated from soil in 1889 and established within the family *Micrococcaceae* in 1947 (Conn and Dimmick, 1947; Koch et al., 1995). *Arthrobacter* have been widely isolated from diverse environments including air, soil, and fresh water. They were recognized for nutritional versatility with respect to the decomposition of organic matter and environmental pollutants (Katznelson and Sirois, 1961; Gunner, 1963). Specific reports of the metabolic versatility of *Arthrobacter* strains have detailed their ability of degrade xenobiotics, such as pesticides, haloalkanes, polycyclic aromatic compounds, and benzene derivatives (Chauhan et al., 2000; Rong et al., 2009; Husserl et al., 2010; Wang et al., 2012; Yao et al., 2015).

In our previous study, a versatile aromatic-degrading bacterium, *Arthrobacter* sp. YC-RL1, was isolated from petroleum contaminated soil, and its genome was sequenced and published, but without further analysis (Ren et al., 2016a,b). In this study, the metabolic versatility of strain YC-RL1 was investigated by: (i) analyzing its ability to degrade seven aromatic compounds, (ii) detection of the intermediates produced during metabolism, and (iii) investigating the genetic basis likely to encode the observed activities. Our results revealed that: (I) strain YC-RL1 could utilize PX, PNP, naphthalene, phenanthrene, biphenyl and BPA for growth, separately and simultaneously, (II) the metabolic pathways of selected aromatic compounds were deduced via metabolic intermediates analysis which suggested that strain YC-RL1 possesses systematic metabolic pathways toward aromatic compounds, (III) several genes and gene clusters (including a novel gene cluster encoding the transformation of biphenyl to benzoate) contributed to the utilization of these aromatic compounds were identified via comparative genomic analysis, and the activity of a 2,3-dihydroxybiphenyl 1,2-dioxygenase was confirmed via heterologous expression.

## Materials and Methods

### Bacterial Strains, Growth Conditions, and Chemicals

*Arthrobacter* sp. YC-RL1 was grown in Luria-Bertani (LB) medium and mineral salt medium (MSM) with PNP or other aromatic compounds at 30°C. Strain YC-RL1 is available from China General Microbiological Culture Collection Center (CGMCC) with accession number CGMCC 10611. *Escherichia coli* DH5α and *E. coli* BL21 were used for gene cloning and gene expression, respectively (the detail information was presented in **Supplementary Table S2**).

The detailed information about aromatic compounds and other reagents used in this study was presented in **Supplementary Table S3**. All the substrates were dissolved in methanol as stock solution (2 × 10^4^ mg/L) separately, sterilized by membrane filtration (0.22 μm), and then supplemented into MSM to obtain the target concentration. LB and MSM media were prepared as previously described (Ren et al., 2016a).

### Biodegradability of Different Aromatic Compounds

An inoculum of strain YC-RL1 was prepared as previously described and then used to determine the biodegradability of different aromatic compounds (Ren et al., 2016a). Two series of measurements were performed: (i) 200 μL of inoculum were added into 20 mL MSM medium supplemented with one of the following substrates: PX, PNP, naphthalene, phenanthrene, biphenyl, BPA or di 2-ethyl hexyl phthalate (DEHP) (50 mg/L, respectively), and cultures without inoculation were set as control; (ii) the substrates above were simultaneously added into 20 mL MSM medium to 50 mg/L each (inoculation of 200 μL inoculum was set as treatment and culture without inoculation was set as control). Meanwhile, inoculation of 200 μL inoculum with 25 μL menthol in 20 mL MSM medium was performed as control too.

Cells were cultivated at 30°C and 180 rpm. After 5 days incubation, samples were extracted twice by an equal volume of ethyl acetate. Extracts were dried by nitrogen-blow and finally dissolved in methanol (equal to the initial volume). The residual concentration of each substrate was then quantified by high-performance liquid chromatography–mass spectrometry (HPLC–MS) analysis. The percent recovery of each substrate was also measured (all above 98.0%). All the experiments were conducted in triplicate.

The calculation of degradation rates described as below:

(1) $$\mathrm{Degradation rate}\;(\%)=\frac{C_{\text{ck}}-C_{\text{f}}}{C_{\text{ck}}}\times 100$$

where *C*_ck_ represents the final concentration of each substrate in the control treatment, *C*_f_ represents the final concentration of each substrate with inoculation of strain YC-RL1.

### Identification of Metabolic Pathways

Naphthalene, BP, BPA, and PNP were selected as representative substrates for further analysis. Strain YC-RL1 was precultured as previously described (Ren et al., 2016a) and inoculated into fresh MSM supplemented with one of the selected substrates (50 mg/L) as sole carbon source. All cultures were cultivated under 30°C and 180 rpm. Subcultures were withdrawn every 12 h and extracted as described above.

The quantification of selected aromatic compounds and identification of metabolic intermediates were accomplished by HPLC–MS analysis. A triple quadrupole mass spectrometer (Agilent 6420, United States) equipped with an electrospray ionization (ESI) was employed in this study. Samples were directly injected into detector (10 μL) and the mobile phase was methanol (100%, 0.2 mL/min). The scan mode with a capillary voltage of 3.5 kV for negative ionization was applied and the carrier gas (nitrogen, 99.999%) was heated to 325 (gas flow: 8 L/min). Negative ions were acquired from 100 to 600 Da and analyzed by Mass hunter (version A.02.00, Agilent, United States).

In order to distinguish some easily confused intermediates (benzoate vs. salicylaldehyde and protocatechuate vs. gentisate), standard chemicals were applied to HPLC analysis. An Agilent 1260 system equipped with an Eclipse Plus C18 column (4.6 × 75 mm × 3.5 μm), a column oven, a quaternary pump, a mixer and a diode array detector was used. The mobile phase was composed of methanol and 50 mM acetic acid solution (pH = 3.0) with a constant ratio (9:1) and the flow rate was 2 mL/min. The injection volume was 20 μL.

Based on detected metabolic intermediates and known reports, the metabolic pathways of selected aromatic compounds were deduced. The multiple interrelated metabolic pathways of different aromatic compounds were built thereafter.

### Genes and Gene Clusters Involved in Aromatics Metabolism

The whole-genome sequencing of strain YC-RL1 was accomplished using PacBio RS II platform to provide further insights into molecular mechanisms of metabolic versatility. The genome of strain YC-RL1 was annotated and deposited into GenBank. The circular representation of genome and plasmids was generated by circos (version 0.69) (Krzywinski et al., 2009). The genome circular comparison between strain YC-RL1 and 5 *Arthrobacter* strains (including three aromatic compounds-degrading strain) were performed using BLAST Ring Image Generator (BRIG, version 0.95) (Alikhan et al., 2011). A summary of genome features of strain YC-RL1 and five other *Arthrobacter* strains were shown in **Supplementary Table S4**. Further, nucleotide-based genome alignment between YC-RL1 and the *Arthrobacter* sp. RE117 was performed to study genome rearrangements using the progressive MAUVE algorithm in the MAUVE genome alignment software (version 2.3.1) (Darling et al., 2010).

For the decomposition of aromatic compounds under oxic conditions, ring-cleavage is known as the key step which is mostly mediated by dioxygenases (Vaillancourt et al., 2008). Based on obtained genome sequence and annotation results, all dioxygenase coding sequences were retrieved and manually screened to identify dioxygenases involved in the catabolism of aromatic compounds. Basic Local Alignment Search Tools (BLAST) of NCBI^1^ was used to locate the target sequences (dioxygenase genes). Their genomic neighborhood were further analyzed to identify possible gene clusters. The identified genes and gene clusters were also further analyzed by RAST web service^2^ to show their similarity with known genomes (Overbeek et al., 2013; Brettin et al., 2015).

The sequence alignment was performed with Clustal X2 program (version 2.1) (Larkin et al., 2007). Phylogenetic relationship of dioxygenases was demonstrated by MEGA 7.0 using neighbor-joining method (the bootstrap value was set as 1000) (Kumar et al., 2016). The genome sequence of strain YC-RL1 has been deposited into GenBank under accession number NZ_CP013297 and also available from RAST database with accession number 1654525.

### Characterization of 2,3-Dihydroxybiphenyl 1,2-Dioxygenase (BphC)

Bacterial strains, plasmids and primers for the amplification and expression of *bphC* were listed in **Supplementary Table S2**. The total DNA of strain YC-RL1 was extracted by Bacterial Genomic DNA Extraction kit (Takara, Japan). The targeted gene was obtained by PCR. The *Ex Taq*^®^ DNA Polymerase (Takara, Japan) was used for the PCR following the protocol. The obtained fragments were inserted into pMD19-T vector and then transformed into *E. coli* DH5α competent cell. Positive transformants were selected for plasmid extraction. The recombinant plasmid was sequenced by Sangon Biotech Co., Ltd. For the gene expression, the target sequence was inserted into pET32a(+) vector and then transformed into *E. coli* BL21. Positive transformants were confirmed by sequencing and then cultured in LB media at 37°C. Targeted protein was induced by isopropyl-D-thiogalactopyranoside (IPTG). The cells were harvested by centrifuging at 10,000 rpm for 3 min, washed, and resuspended in the PBS buffer (pH 7.8). The cell suspension was disrupted by sonication and centrifuged at 8,000 rpm for 5 min to obtain the supernatant. The targeted protein was purified from the supernatant by immobilized metal ion affinity chromatography (ÄKTA, GE Healthcare, United States). The purification was confirmed by SDS-PAGE. Finally, the purified protein was used for functional confirmation. The function of BphC was confirmed by measuring the transformation of 2,3-dihydroxy biphenyl, briefly: (i) monitoring the products 2-hydroxy-6-oxo-6-phenylhexa-2,4-dienoate (HOPDA) at 434 nm with a scanning spectrophotometer, (ii) observing the change color of the reaction mixture, (iii) analyzing the generated products by HPLC-MS.

## Results

### Biodegradability of Different Aromatic Compounds

**FIGURE 1:**
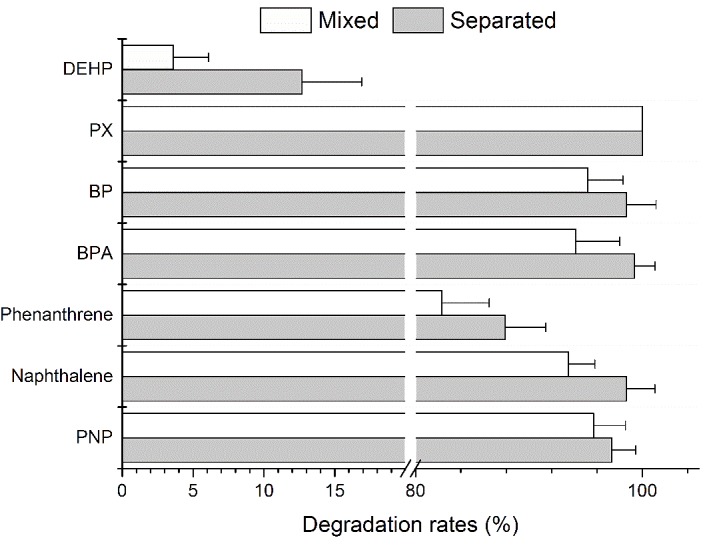
The degradation rates of different aromatic compounds under separated and mixed situations.

The degradation rates of selected aromatic compounds, alone and as part of a mixture, is shown in **Figure 1**. PX was completely degraded when alone and as part of a mixture. The percent removal of PNP, naphthalene, BPA and biphenyl were all above 90.0% under both situations. The percent removal of phenanthrene was 87.9 ± 3.6% and 82.3 ± 4.2% when alone and as part of a mixture, respectively. However, the degradation rates of DEHP was very low and no cell growth was observed which indicated that strain YC-RL1 could not utilize DEHP as carbon source for growth. However, very little DEHP was degraded (12.7 ± 4.1% and 3.6 ± 2.5% under separated and mixed conditions, respectively) due to fortuitous hydrolysis by broad-spectrum hydrolases. These results demonstrated that strain YC-RL1 could utilize different kinds of aromatic compound for growth. As to the inability of DEHP utilization, the lack of necessary hydrolases might be the reason. The decomposition of DEHP consists two main steps: transformation of DEHP into benzoate and utilization of benzoate (Net et al., 2015; Ren et al., 2018). Two ester bond hydrolases, DEHP hydrolase and mono-(2-ethylhexyl) phthalate (MEHP) hydrolase, contributed to the first step (Latorre et al., 2012; Nahurira et al., 2017). These results also indicated that strain YC-RL1 possess systematic metabolic pathways for aromatic compounds and related molecular mechanism.

### Identification of Metabolic Pathways

**FIGURE 2:**
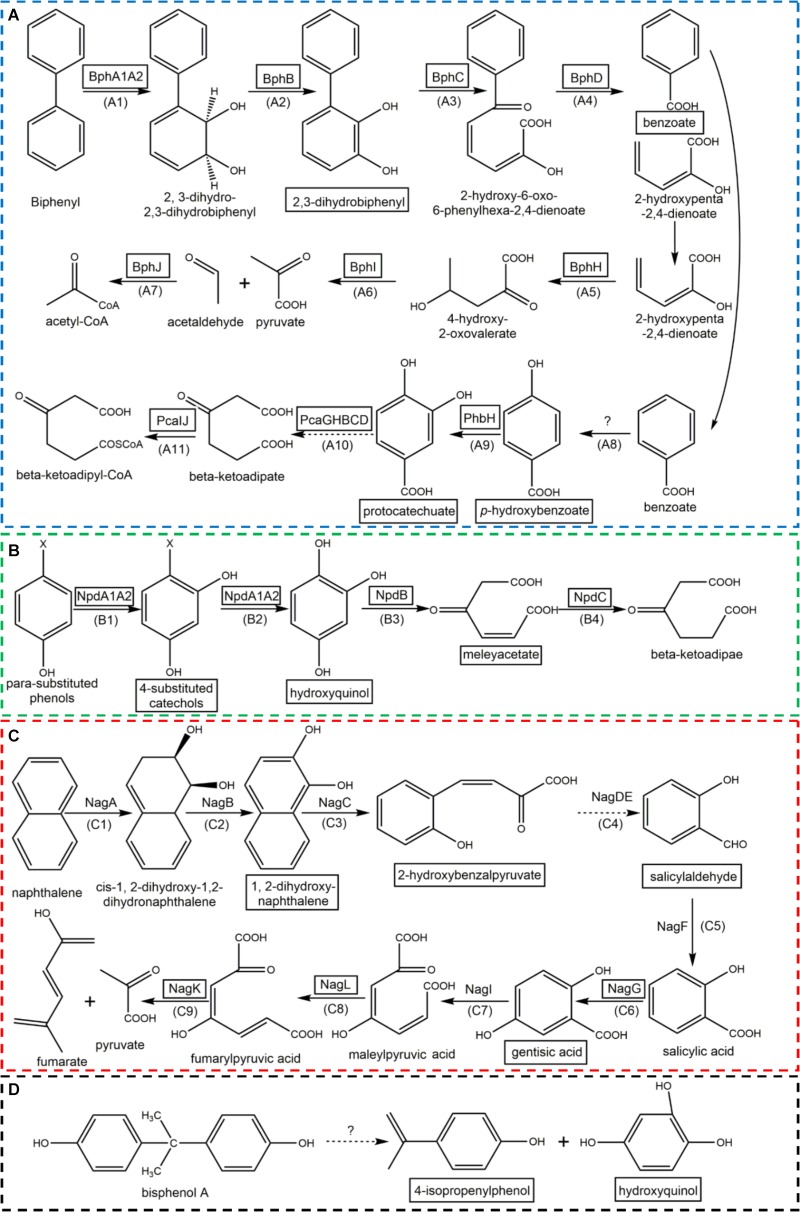
The metabolic pathways of biphenyl **(A)**, para-substituted phenols **(B)**, naphthalene **(C)**, and bisphenol A **(D)**. (Enzymes in block diagram indicate that they were identified in strain YC-RL1; metabolic intermediates in block diagram indicate that they were detected by HPLC-MS; the dotted arrows mean there are more than one enzyme contributed to this process; the detailed information of each step (under each arrow) were described in **Table 2**; ‘?’ means the enzymes involved in were still unknown).

On the basis of HPLC and HPLC-MS analysis (**Supplementary Figure S1**) and known reports, the potential metabolic intermediates of PNP, naphthalene, biphenyl and BPA were listed in **Table 1**. Detailed information of detected substances, including *m/z*, proposed intermediates, molecular formula and molecular weight, were presented too. Some intermediates were simultaneously detected in the degradation of two or more kinds of aromatic compounds (**Table 1**, marked with gray background), including protocatechuate, hydroxyquinol, and maleylacetate. Given the huge variety of aromatic compounds, it is not surprising that there is a diversity of metabolic pathways for the degradation of these compounds. As reviewed by Fuchs et al. (2011), microbes transform different aromatic compounds via a limited number of intermediates, for example, benzoate, hydroxyquinol, and protocatechuate, which are then susceptible to ring-cleavage reactions. The metabolic pathways of selected aromatic compounds were deduced according to the obtained intermediates information (**Figure 2**).

**Table 1:** The metabolic intermediates of aromatic compounds.

Substrates	No.	*m/z*	Proposed intermediates	M.F.	M.W.
Biphenyl	1	185.2	2,3-Dihydroxybiphenyl	C_12_H_10_O_2_	186.21
	2	121.1	Benzoate	C_7_H_6_O_2_	122.12
	3	153.1	Protocatechuate	C_7_H_6_O_4_	154.12
Naphthalene	4	159.1	1,2-Dihydroxynaphthalene	C_10_H_8_O_2_	160.17
	56	191.1	*cis*-*o*-Hydroxybenzalpyruvate	C_10_H_8_O_4_	192.17
	7	121.1	Salicylaldehyde	C_7_H_6_O_2_	122.12
		153.1	Gentisic acid	C_7_H_6_O_4_	154.12
Bisphenol A	8 9	133.2	4-Isopropenylphenol	C_9_H_10_O	134.19
	10	109.1	Hydroquinone	C_6_H_6_O_2_	110.11
	11	137.1	4-Hydroxybenzoic acid	C_7_H_6_O_3_	138.12
	12	153.1	Protocatechuate	C_7_H_6_O_4_	154.12
	13	125.1	Hydroxyquinol	C_6_H_6_O_3_	126.11
		157.1	Maleylacetate	C_6_H_6_O_5_	158.11
*p*-Nitrophenol	14	154.1	4-Nitrocatechol	C_6_H_5_NO_4_	155.11
	15	125.1	Hydroxyquinol	C_6_H_6_O_3_	126.11
	16	157.1	Maleylacetate	C_6_H_6_O_5_	158.11

*M.F., molecular formula; M.W., molecular weight.*

For the metabolism of biphenyl (**Figure 2A**), biphenyl was transformed into benzoate and 2-hydroxypenta-2,4-dienoate via 2,3-dihydroxybiphenyl (2,3-DHBP, through ring hydroxylation) and HOPDA (through ring-cleavage). Benzoate was then transformed into protocatechuate and further utilized through ring-cleavage (benzoate pathway). The metabolism of PNP (**Figure 2B**) belongs to well-known 4-nitrocatechol (4-NC) pathway since PNP was transformed into hydroxyquinol via 4-NC. Then, hydroxyquinol entered the hydroxyquinol pathway. The degradation of naphthalene (**Figure 2C**) was similar with the process of biphenyl. Firstly, naphthalene was transformed into salicylaldehyde via 1,2-dihydroxynaphthalene. Salicylic acid was then generated with the dehydrogenation of salicylaldehyde. Salicylic acid was hydroxylated into gentisic acid. The ring-cleavage of gentisic acid formed maleylpyruvic acid which was then transformed into fumarate and pyruvate via fumaryl-pyruvic acid. For the metabolism of BPA (**Figure 2D**), hydroxyquinol and 4-isopropenylphenol were identified which indicated that two aromatic rings were separated before ring-cleavage. Hydroxyquinol was utilized via hydroxyquinol pathway. 4-Isopropenylphenol was transformed into protocatechuate via 4-hydroxybenzoate and then entered benzoate pathway.

### Genes and Gene Clusters Contributed to the Metabolic Versatility

**FIGURE 3:**
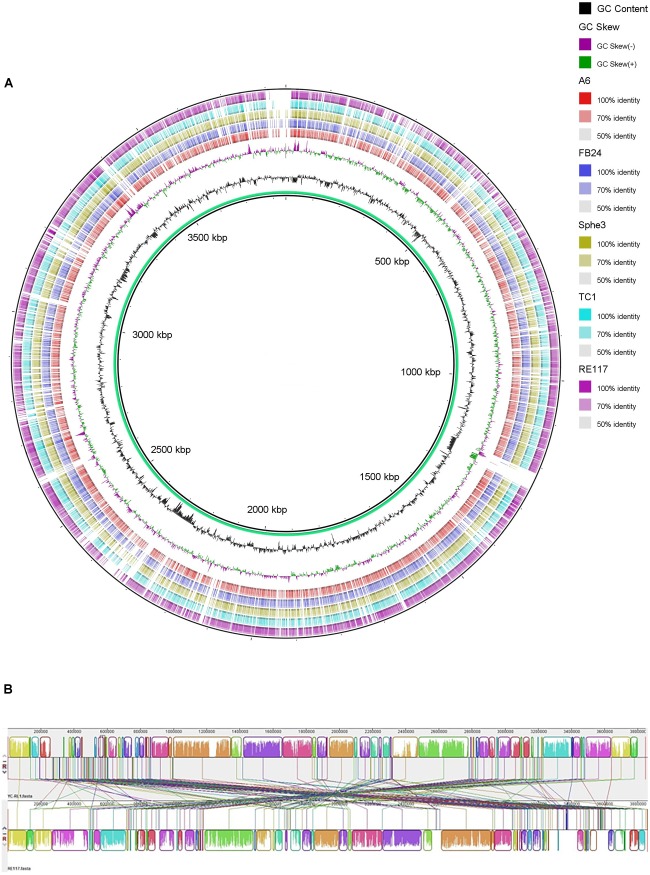
Whole-genome comparisons of strain YC-RL1 with five *Arthrobacter* strains **(A)** and nucleotide-based genome alignment between strain YC-RL1 and RE117 **(B)**. [**(A)** The color intensity in each ring represents the BLAST match identity. **(B)** Homologous blocks were presented with identically colored regions and linked across the sequences].

The genome of strain YC-RL1 is consisted of a circular chromosome (3,846,272 base pairs with an average G + C content of 64.24%) and two plasmids [plasmid 1 (112,763 bp, G + C = 57.81%) and plasmid 2 (59,604 bp, G + C = 58.96%)] (**Supplementary Figure S2**). A visual inspection of the circular alignment of genomes highlighted sequence similarity between query genomes and reference genome (**Figure 3A**) and the results indicated that strain YC-RL1 showed high genome similarity with *Arthrobacter* sp. RE117. The chromosomal alignments between strain YC-RL1 and RE117 indicated that the genome structures between two strains were almost identical (**Figure 3B**). A total of 170 homologous locally collinear blocks (LCBs) were recognized and these blocks sequentially located on the genome.

Genome annotation and comparative genomics analysis of YC-RL1 revealed a variety of genes involved in the metabolism of aromatic compounds (**Supplementary Dataset S1**). A total of eight dioxygenase genes were found in the genome and plasmids, three of which had homology to ring-cleavage dioxygenase genes (**Supplementary Table S5**).

**FIGURE 4:**
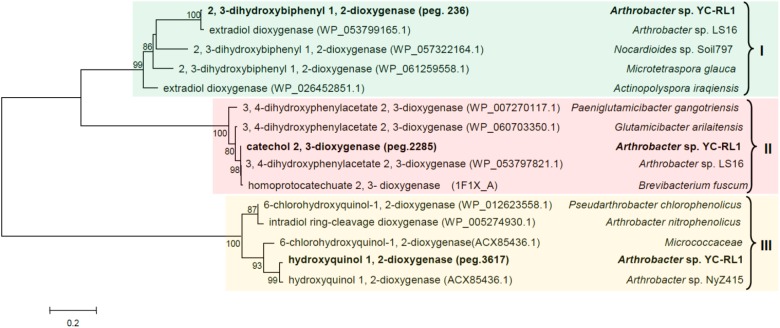
Phylogenetic tree of three ring-cleavage dioxygenases in YC-RL1 with known ring-cleavage dioxygenases. (Three different colors suggested three different categories of dioxygenase; type I and type II are extradiol dioxygenase, type III is intradiol dioxygenase; the accession numbers of each sequence were included in the parentheses. The phylogenetic tree was constructed by MEGA 7.0 software using neighbor-joining method and the bootstrap value was set as 1000. Gaps and hypervariable regions were remained for the final alignment and the number of positions used for the final alignment is 381. The scale bar represents the number of expected changes per site).

Typically, there are two types of dioxygenase involved in the ring-cleavage of aromatic compounds, intradiol and extradiol dioxygenases (Wang et al., 2017). The diversity of dioxygenases significantly affects the metabolic versatility because the substituted groups on the benzene ring decide the position of ring-cleavage and potential toxicity of the substrate to the enzyme itself. To have an in-depth understanding of the metabolic versatility, three ring-cleavage dioxygenases were selected for further analysis. Reported ring-cleavage dioxygenases were downloaded from GenBank. As shown in **Figure 4**, both intradiol and extradiol ring-cleavage dioxygenases were detected in YC-RL1. As the substituted groups on the benzene ring decide the position of ring-cleavage, the diversity of ring-cleavage dioxygenases partially explained the metabolic versatility of strain YC-RL1.

Genes flanking ring-cleavage dioxygenase genes were searched and conserved clusters were identified by RAST server (**Supplementary Dataset S2**). All the identified genes and gene clusters were manually checked. Three conserved gene clusters involved in aromatic-degradation were identified by comparative genomics analysis, including *npd* (encoding para-substituted phenols metabolic pathway), *bph* (a novel gene cluster encoding biphenyl degradation), and *pca* (encoding protocatechuate branch of β-ketoadipate pathway) (**Table 2**) (Perry and Zylstra, 2007; Liu et al., 2010; Pham and Sylvestre, 2013). The sequence similarities of *npd* and *pca* genes with known reports were all above 90%. As the *pca* gene cluster has been extensively investigated, we would not describe and discuss it here (Harwood and Parales, 1996).

**Table 2:** Enzymes involved in the metabolism of biphenyl, protocatechuate, para-substituted phenols, and naphthalene.

Function	Steps	Enzymes	E. C. number	Genes (Accession numbers)	Gene cluster
Metabolism of biphenyl	A1	Biphenyl dioxygenase	1.14.12.18	*bphA1* (peg.242), *bphA2* (peg.240)	*bph*
	A2	2,3-Dihydroxy-1-phenylcyclohexa-4,6-diene dehydrogenase	1.3.1.56	*bphB* (peg.243)	
	A3	2,3-Dihydroxybiphenyl 1,2-Dioxygenase	1.13.11.39	*bphC* (peg.236)	
	A4	2-Hydroxy-6-oxo-6-phenylhexa-2,4-dienoic acid hydrolase	3.7.1.9	*bphD* (peg.239)	
	A5	2-Keto-4-pentenoate hydratase	4.2.1.80	*bphH* (peg.303)	
	A6	4-Hydroxy-2-oxovalerate aldolase	4.1.3.39	*bphI* (peg.301)	
	A7	Acetaldehyde dehydrogenase	1.2.1.10	*bphJ* (peg.302)	
Metabolism of protocatechuate	A9	*p*-Hydroxybenzoate hydroxylase	1.14.13.2	*phbH* (peg.3194)	*pca*
	A10	Protocatechuate 3,4-dioxygenase	1.13.11.3	*pcaG* (peg.3196), *pcaH* (peg.3195)	
	A10	3-Carboxy-*cis*, *cis*-muconate cycloisomerase	5.5.1.2	*pcaB* (peg.3197)	
	A10	4-Carboxymuconolactone decarboxylase	4.1.1.44	*pcaC* (peg.3199)	
	A10	Beta-ketoadipate enol-lactone hydrolase	3.1.1.24	*pcaD* (peg.3198)	
	A11	3-Ketoacid-CoA transferase	2.8.3.6	*pcaI* (peg.3188), *pcaJ* (peg.3187)	
	/	3-Ketoacyl-CoA thiolase	2.3.1.16	*pcaF* (peg.3189)	
	/	*pca* regulon regulatory protein	/	*pcaR* (peg.3186)	
	/	Benzoate MFS transporter	/	*benK* (peg.3190)	
Metabolism of para-substituted phenols	B1	Para-substituted phenols monooxygenase	1.14.13.166	*npdA1* (WP_047117614), *npdA2* (peg.3615)	*npd*
	B2	Hydroxyquinol 1,2-dioxygenase	1.13.11.37	*npdB* (peg.3617)	
	B3	Maleylacetate reductase	1.3.1.32	*npdC* (WP_047117615)	
	B4	Regulatory protein	/	*npdR* (peg.3616)	
Metabolism of naphthalene	C1	Naphthalene 1,2-dioxygenase	1.14.12.12	*nagA*	*nag*
	C2	Dihydroxynaphthalene dehydrogenase	1.3.1.29	*nagB*	
	C3	1,2-Dihydroxynaphthalene dioxygenase	1.13.11.56	*nagC*	
	C4	2-Hydroxychromene-2-carboxylate isomerase	5.99.1.4	*nagD*	
	C4	*Trans*-*o*-hydroxybenzylidenepyruvate hydratase-aldolase	4.1.2.45	*nagE*	
	C5	Salicylaldehyde dehydrogenase	1.2.1.65	*nagF*	
	C6	Salicylate-5-hydroxylase	1.14.13.172	*nagG* (peg.576)	
	C7	Gentisate 1,2-dioxygenase	1.13.11.4	*nagI*	
	C8	Maleylpyruvate isomerase	5.2.1.4	*nagL* (peg.2472)	
	C9	Fumarylpyruvate hydrolase	3.7.1.2	*nagK* (peg.724)	

*Steps are correlated with **Figure 2**; ^∗^ genes without accession number mean they were not identified in the genome of strain YC-RL1; ‘/’ means not accessible.*

**FIGURE 5:**
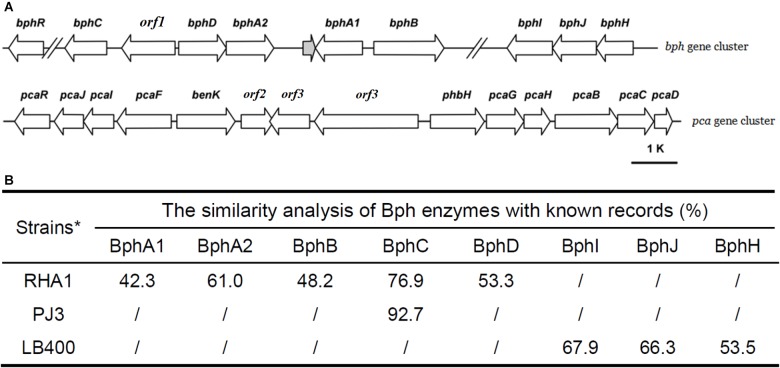
The genetic organization of *bph* and *pca* gene cluster **(A)** and the similarity analysis of Bph enzymes with known records **(B)**. [‘//’ means a long distance between two genes; the distance between *bphC* and *bphR* is 7192 bp; the distance between *bphB* and *bphI* is 62,400 bp; RHA1: *Rhodococcus* sp. RHA1 (Masai et al., 1997), LB400: *Pseudomonas* sp. LB400 (Hofer et al., 1994)].

The complete metabolic pathway of biphenyl includes three main steps (**Figure 2**): (I) transformation of biphenyl into 2,3-dihydroxybiphenyl, in which O_2_ was introduced at the 2,3-position of biphenyl to produce dihydrodiol and then dehydrogenated to 2,3-dihydroxybiphenyl; (II) ring-cleavage of 2,3-dihydroxybiphenyl and then the hydrolysis of the product (HOPDA) to produce benzoate and 2-hydroxypenta-2,4-dienoate; (III) utilization of benzoate and 2-hydroxypenta-2,4-dienoate. Based on homology to well characterized biphenyl degradation genes, three gene clusters were identified in the genome of strain YC-RL1 that are likely to encode the complete degradation of biphenyl (**Figure 5A**: *bphA1A2BCD*, *bphHIJ*, and *pcaGHBCD*). The homology analysis of Bph enzymes were performed with known records (**Figure 5B**) and the results indicated that gene cluster *bphA1A2BCD* was a novel biphenyl-degrading gene cluster. This novel gene cluster encodes enzymes initialed the degradation of biphenyl, including a biphenyl dioxygenase genes (*bphA1A2*), a dihydrodiol dehydrogenase gene (*bphB*), a 2,3-dihydroxybiphenyl dioxygenase gene (*bphC*), a HOPDA hydroxylase gene (*bphD*), and a potential transcriptional regulator gene (*bphR*) (**Figure 5A**). Gene cluster *bphHIJ* was identified next to *bphA1A2BCD* and likely encodes for the transformation of 2-hydroxypenta-2,4-dienoate into acetyl-CoA. For the utilization of benzoate, one widely reported *pca* gene cluster was identified and located on the chromosome. Benzoate was utilized via protocatechuate branch of β-ketoadipate pathway, which was encoded by *pcaGHBCD*. Genes (*pcaIJF*), responsible for the utilization of β-ketoadipate, were also identified in *pca* gene cluster. Meanwhile, *p*-hydroxybenzoate hydroxylase gene (*phbH*) and benzoate MFS transporter gene (*benK*) were also identified in *pca* gene cluster. This is the first report of the complete biphenyl metabolic pathway and related genes in genus *Arthrobacter*.

**FIGURE 6:**
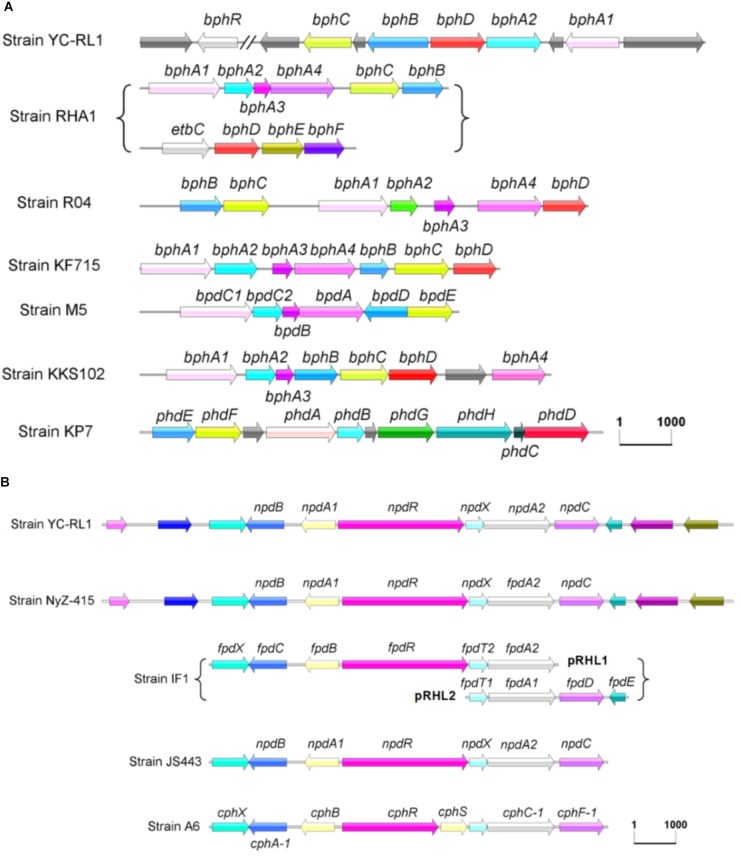
Gene clusters involved in the degradation of biphenyl **(A)** and para-substituted phenols **(B)**. (Strain RHA1: *Rhodococcus* sp. RHA1, Strain R04: *Rhodococcus* sp. R04, Strain KF715: *Pseudomonas putida* KF715, Strain M5: *Rhodococcus* sp. M5, Strain KKS102: *Achromobacter georgiopolitanum* KKS102, Strain KP7: *Nocardioides* sp. KP7, Strain NyZ-415: *Arthrobacter* sp. NyZ415, Strain IF1: *Arthrobacter* sp. IF1, Strain JS443: *Arthrobacter* sp. JS443, Strain A6: *Arthrobacter chlorophenolicus* A6).

There are three key steps in the degradation of para-substituted phenols (**Figure 2B**): (i) addition of hydroxyl groups to the substrates, (ii) cleavage of the aromatic ring, and (iii) reduction of maleylacetate. In strain YC-RL1, three enzymes and one transcription regulator involved in the degradation of PNP were identified. Known gene clusters (in genus *Arthrobacter*) involved in the degradation of para-substituted phenols were presented in **Figure 6A**. The degradation of para-substituted phenols was initialed by a two-component monooxygenase (NpdA1 and NpdA2, reductase and oxygenase components, respectively). This monooxygenase system is a typical class D flavoprotein monooxygenases (two-component flavin-diffusible monooxygenase, TC-FDM). The reductase component (NpdA1) of monooxygenase is able to reduce FAD/FMN at the expense of NAD(P)H. NADH is the preferred electron donor and FAD is the preferred electron acceptor. Then, the oxygenase component (NpdA_2_) of monooxygenase receives electrons from the FADH_2_ and hydroxylates the substrates with O_2_. The generated hydroxyquinol is further applied for ring-cleavage which is mediated by a hydroxyquinol 1,2-dioxygenase (NpdB). The product (maleyacetate) is further hydrolyzed to β-ketoadipate and then enters the TCA cycle.

For the degradation of naphthalene, genes contributed to the peripheral pathway were not found and three genes (*nagG*, *nagL*, and *nagK*) involved in the utilization of salicylic acid were identified. Same situation occurred with BPA degradation and one cytochrome P450 protein (peg.429) potentially involved in the degradation of BPA was identified, which could transform BPA into 1, 2-bis(4-hydroxyphenyl)-2-propanol and 2,2-bis(4-hydroxyphenyl)-1-propanol. The similarity of the identified cytochrome P450 protein with known BPA-degrading cytochrome P450 protein (BAG15884) is 47% (Sasaki et al., 2008). Other enzymes related to BPA metabolism are still unknown and further investigation is needed.

### Functional Confirmation of 2,3-Dihydroxybiphenyl 1,2-Dioxygenase (BphC)

**FIGURE 7:**
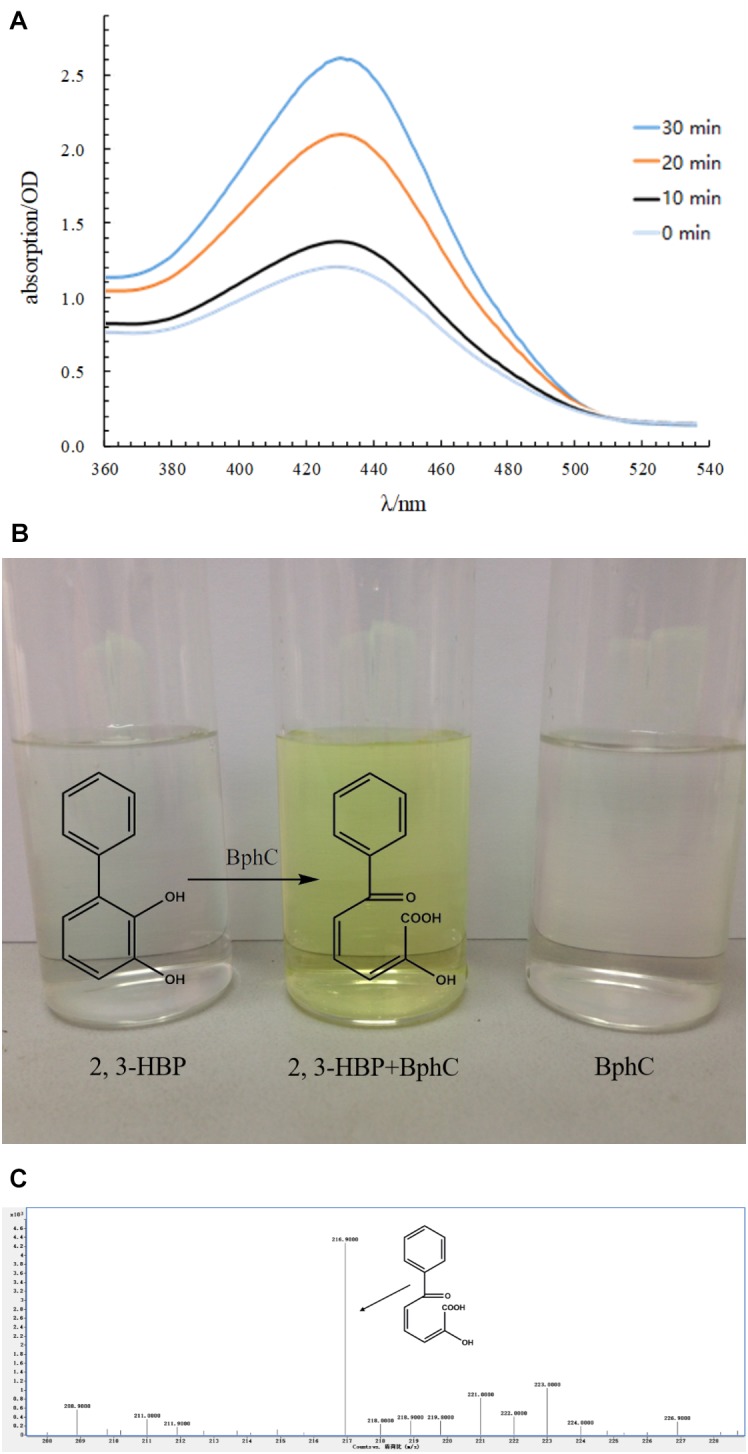
Functional characterization of BphC. (**A**: Monitoring the production of HOPDA using a scanning spectrophotometer; **B**: Reaction of BphC and 2,3-dihydroxy biphenyl (2,3-HBP); **C**: HPLC-MS analysis of the yellow products).

The *bphC* gene with an open reading frame of 930 bp was cloned and inserted into a pET32a(+) vector. The expression and purification of BphC was confirmed by SDS-PAGE (**Supplementary Figure S3**). The calculated molecular weight (M.W.) of BphC is 34.14 kDa and the tagged-sequence has a M.W. of approximately 20 kDa. The recombinant BphC protein with tagged-sequence was successfully purified and appeared as a 54 kDa protein on the SDS gel. The purified BphC was applied to the transformation of 2,3-dihydroxybiphenyl. The activity was measured by monitoring the products (HOPDA) with a scanning spectrophotometer. The specific absorption of HOPDA was observed at 434 nm and the maximum absorption value was at 30 min. Results shown that, the absorption values at 434 nm increased with the time (**Figure 7A**). That means the concentration of HOPDA increased with the reaction. BphD was able to degrade 86.3 ± 4.2% of 50 mg/L 2,3-dihydroxybiphenyl in 30 min at 25°C accompanied with color change (colorless to yellow, **Figure 7B**). The generated yellow products were further analyzed by HPLC-MS and showed a *m/*z of 217 (M.W. = 218) which suggested the yellow product was HOPDA (**Figure 7C**).

## Discussion

The metabolic versatility of microbes is an important characteristic of their role in the cycling of matter and the detoxification of hazardous compounds (Larimer et al., 2003; Llamas et al., 2006). Most reports about genus *Arthrobacter* have focused on their role in the metabolism of xenobiotics, especially benzenoids and their derivatives, as well as heterocyclic compounds and hydrocarbons. For example, nicotine degrading strain M2012083 was isolated from tobacco waste and identified as *Arthrobacter aurescens*. The comparative genome analysis of strain M2012083 revealed the molecular basis of nicotine degradation and survival capacities of *Arthrobacter* species (Yao et al., 2015). Many *Arthrobacter* species were found to be involved in the catabolism of substituted phenols, such as 4-nitrophenol, 4-chlorophenol, and 4-fluorophenol (Nordin et al., 2005; Perry and Zylstra, 2007; Ferreira et al., 2009). For strain YC-RL1, the efficient utilization of several kinds of aromatic compounds was confirmed which suggested its metabolic versatility and application potential.

As to the failure of DEHP utilization, the lack of necessary esterase might be the reason. To verify this hypothesis, two works were further conducted: (i) all the annotations of hydrolase and esterase were manually checked to identify potential enzymes involved in the hydrolysis of ester bond in DEHP; (ii) the sequences of all reported esterase/hydrolase involved in the hydrolysis of phthalic acid esters (PAEs) were downloaded and searched against the genome of strain YC-RL1 to identify related enzymes. All annotated esterases/hydrolases were shown in **Supplementary Dataset S3**. A total of 33 hydrolases and 8 esterases were identified and only 7 hydrolases were related to metabolism of aromatic compounds. All these esterases and hydrolases were manually checked by BLAST and the results indicated that none of these was related to the metabolism of DEHP or PAEs. As reviewed by Ren et al. (2018), 14 esterases/hydrolases, belonging to four families of esterase and one unknown family, were reported to be involved in the metabolism of PAEs until now. All these 14 esterases/hydrolases were searched against the genome of strain YC-RL1 and no positive result was obtained. Meanwhile, the reports of *Arthrobacter* isolates with the ability of DEHP (or PAEs) utilization were limited too (Chatterjee and Dutta, 2008; Wen et al., 2014). These indicated that the lack of esterase (which contributed to the hydrolysis of ester bonds in PAEs) in genus *Arthrobacter* might be a general situation.

As described by Fuchs et al. (2011), biodegradation of aromatic compounds is typically divided into two main steps, peripheral (upper) pathways and central (lower) pathways: microbes metabolize a diversity of aromatic compounds via peripheral pathways into a few key common intermediates such as catechol, protocatechuate and benzoate, etc. Central pathways then cleave the aromatic ring and convert these intermediate products into central metabolites such as acetyl-CoA, succinyl-CoA and pyruvate, which can be used for microbial growth. This metabolic convergence is a typical and useful strategy for microbial growth that limits the genetic burden necessary to encode the metabolism of a vast array of natural and man-made products. In strain YC-RL1, different kinds aromatics were transformed into several kinds of metabolic intermediates (salicylic acid, protocatechuate, hydroxyquinol, etc.) via different peripheral pathways. As to the central pathway, the utilization of these intermediates has been widely and systematically investigated such as benzoate pathway, hydroxyquinol pathway, and beta-ketoadipate pathway.

Based on the whole-genome analysis, three dioxygenases involved in the ring-cleavage were identified, including both intradiol and extradiol dioxygenases. Gene clusters contributed to the catabolism of para-substituted phenols, biphenyl, and protocatechuate were identified. Of these, gene cluster *bphA1A2BCD* (contributed to the peripheral pathway of biphenyl catabolism) was a novel biphenyl-degrading gene cluster.

For the degradation of para-substituted phenols, *Arthrobacter* was known as one of the predominant degraders. The degradation pathways have been proposed in some isolated strains, including *Arthrobacter* sp. IF1 (4-fluorophenol), *Arthrobacter* sp. JS443 (4-nitrophenol), *Arthrobacter chlorophenolicus* A6 (4-chlorophenol and 4-nitrophenol), and *Arthrobacter* sp. NyZ415 (4-nitrophenol) (Nordin et al., 2005; Perry and Zylstra, 2007; Ferreira et al., 2009; Liu et al., 2010). Although the location of gene clusters and the gene names were different, this gene cluster (*npd* gene cluster, **Figure 6A**) is highly conserved and only reported in *Arthrobacter* species. However, some differences exist: (i) this gene cluster was located on genome except for YC-RL1 (plasmid2), (ii) genes in this gene cluster were located in a same region except for strain IF1, in which genes were separated in two regions, and (iii) the transcriptional regulator NpdR in strain YC-RL1 contains a conserved bacterial transcriptional activator domain (BTAD), which belongs to the DNRI/REDD/AFSR family of regulators (obtained from SMART database, data not shown), but the transcriptional regulator in strain A6 was divided into two [CphR (AAN08756) and CphS (AAN08755)]. The identification of *npd* gene cluster explained the degrading ability of para-substituted phenols in strain YC-RL1. Further, we compared the G + C content (mol%) of genome (64.26%), plasmid2 (58.96%) and *npd* gene cluster (59.42%). This indicated that plasmid2, including *npd* gene cluster, was possibly obtained by horizontal transfer. These results may also provide important information to evaluate the origin and evolution of *npd* gene cluster.

Although several *Arthrobacter* strains which could degrade biphenyl have been isolated, the complete gene clusters contributed to the metabolism of biphenyl have not been identified in genus *Arthrobacter*. Péloquin and Greer (1993) obtained the gene cluster (DNA fragment, not the complete sequence information) contributed to the degradation of biphenyl and sequenced the partial sequence of *bphC*. Yang M. et al. (2007) identified and characterized BphC in *Arthrobacter* PJ3. The identification of complete gene clusters involved in the degradation of biphenyl, especially the identification of the novel *bph* gene cluster, would advance the investigation of biphenyl metabolism. And different from *npd* gene cluster, *bph* gene cluster showed high diversity (both sequence similarity and structure of gene cluster, **Figure 6B**), even the catabolic pathways were same.

For the metabolism of biphenyl, a multi-component biphenyl dioxygenase, Rieske-type oxygenase (ROs), initials the process by adding hydroxy group to generate dihydrodiol compound (Colbert et al., 2000). The ROs contains a large group of aromatic ring-hydroxylating dioxygenases, which could stereoselectively introduce two hydroxy groups in one enzymatic step (Fernández de las Heras et al., 2012; Chen et al., 2014; Gally et al., 2015). The ROs is always comprised of an oxygenase (BphA1) and a reductase (BphA2). The oxygenase contains a Rieske domain with a [2Fe–2S] cluster and a catalytic domain with a mononuclear Fe(II) binding site. The Rieske [2Fe–2S] cluster accepts electrons from the reductase and transfers them to the mononuclear iron for catalysis. The product, 2,3-dihydroxy-1-phenylcyclohexa-4,6-diene (dihydrodiol compound), is then dehydrogenated to 2,3-dihydroxybiphenyl by a dehydrogenase. The gene coding for the dehydrogenase varies from different species and shows high similarity with 3-ketosteroid-Δ-dehydrogenase, which is involved in the transformation of steroid compounds (Itagaki et al., 1990; Choi et al., 1995; Fernández de las Heras et al., 2012). The dioxygenases which mediated the ring-cleavage of 2,3-dihydroxybiphenyl at the 1, 2-position shows high similarity. Genes coding for 2,3-dihydroxybiphenyl dioxygenases are always selected as the probe for the detection of *bph* gene cluster and become the most extensively investigated (Hauschild et al., 1996; Sugimoto et al., 1997; Sakai et al., 2002; Yang X. et al., 2007). Then, the product (HOPDA) is hydrolyzed by a C–C bond hydrolase (BphD). The location of the hydrolase gene (*bphD*) varies from different species. In some species, *bphD* is clustered with *bphABC* like *Rhodococcus* sp. R04, *Pseudomonas putida* KF715, *Achromobacter georgiopolitanum* KKS102, *Rhodococcus* sp. M5, and *Nocardioides* sp. KP7 (Kikuchi et al., 1994; Labbe et al., 1997; Nishi et al., 2000; Saito et al., 2000; Yang X. et al., 2007). In some other species, *bphD* is separated from *bphABC* and even locates on different plasmids such as *Rhodococcus* sp. RHA1, one of the most extensively investigated biphenyl degrading strain (Masai et al., 1997). The products of C-C bond hydrolysis, benzoate and 2-hydroxypenta-2,4-dienoate, are further utilized via protocatechuate branch of beta-ketoadipate pathway (benzoate) and BphHIJ mediated pathway (2-hydroxypenta-2,4-dienoate).

In strain YC-RL1, the biphenyl dioxygenase system was consisted of an oxygenase component (BphA1) and a reductase component (BphA2). The conserved domain analysis showed that BphA2 comprised a ferredoxin reductase like oxidoreductase domain and a 2Fe–2S iron–sulfur cluster binding domain. The 2Fe–2S iron–sulfur cluster binding domain transferred electrons from NADH to FAD to form FADH_2_. The electrons in FADH_2_ were further transferred by the ferredoxin reductase like oxidoreductase domain. The oxygenase component (BphA1), containing a Rieske (2Fe–2S) domain, accepted electrons from the reductase component and transferred them to the mononuclear iron for catalysis, which transformed biphenyl into dihydrodiol compound. The dihydrodiol dehydrogenase (BphB) showed high similarity to 3-ketosteroid-Δ^1^-dehydrogenase, which catalyzed the introduction of a double bond into the position of carbon 1 and 2 of ring A of 3-ketosteroid. The dihydrodiol compound was dehydrogenated into 2,3-dihydroxybiphenyl by BphB. Then, 2,3-dihydroxybiphenyl was cleaved at the *meta*- position by a highly conserved dioxygenase (BphC), which belongs to the extradiol dioxygenase. The C–C bond hydrolase (BphD) worked on the meta-cleavage product (HOPDA). The hydrolysis product of HOPDA, benzoate, was further utilized via protocatechuate branch of beta-ketoadipate pathway. The protocatechuate and hydroxyquinol branches of beta-ketoadipate pathways showed significant differences and they are responsible for benzoic and phenolic intermediates. Another hydrolysis product of HOPDA, 2-hydroxypenta-2,4-dienoate, was transformed into 4-hydroxy-2-oxovalerate by a 2-keto-4-pentenoate hydratase (BphH). Then, 2-keto-4-pentenoate was degraded into pyruvate and acetaldehyde via 2-keto-4-pentenoate aldolase (BphI). Acetaldehyde was further transformed into acetyl-CoA by acetaldehyde dehydrogenase (BphJ). Based on the above results, the complete metabolic pathways and enzymes involved in the metabolism of biphenyl were identified via metabolic and genomic analysis.

This work systematically demonstrated the metabolic versatility of aromatic compounds in *Arthrobacter* sp. YC-RL1 via metabolic and genomic analysis. Future characterization of the identified enzymes presented in this work, to understand their function in the metabolism of aromatic compounds, as well as their contribution to the metabolic versatility of strain YC-RL1, will help to the development of efficient strategies for the *in situ* bioremediation of aromatic compounds contaminated sites.

## Author Contributions

LR and YY designed the research. LR, YJ, ZL, and RZ contributed to experimental work. LR, ZZ, and HH finished the data analysis. LR and YY wrote and revised the manuscript.

## Conflict of Interest Statement

The authors declare that the research was conducted in the absence of any commercial or financial relationships that could be construed as a potential conflict of interest.

## Footnotes

1 https://blast.ncbi.nlm.nih.gov/Blast.cgi

2 rast.nmpdr.org
