# Supplementary material for: Insight Into Metabolic Versatility of an Aromatic Compounds-Degrading Arthrobacter sp. YC-RL1
Source: Front Microbiol. 2018 Oct 11;9:2438. doi: 10.3389/fmicb.2018.02438 (PMC6193132; doi:10.3389/fmicb.2018.02438)
Supplement: Supplementary file 1 [file Presentation_1.pdf]

Supplementary materials

Insight into Metabolic Versatility of an Aromatic Compounds-Degrading Arthrobacter sp. YC-RL1

Lei Ren[1,2], Yang Jia[2], , Rui Zhang[1, 3], Zhong Lin[1, 4], Zhen Zhen[1] , Hanqiao Hu[1], Yanchun Yan[2],

1. Agricultural College, Guangdong Ocean University, Zhanjiang 524088, China;
2. Graduate School of Chinese Academy of Agricultural Sciences, Beijing 100081, China;
3. Shenzhen Research Institute of Guangdong Ocean University, Shenzhen 518000, China;
4. Faculty of Chemistry and Environmental Science, Guangdong Ocean University, Zhanjiang 524088, China.

Address correspondence to: Yanchun Yan, Graduate School of Chinese Academy of Chinese Agricultural Sciences, Beijing, 100081, PR China. Tel: +86 10 82109685; Fax: +86 10 82106609; E-mail: yanyanchun2012@qq.cn.

Table S1| Typical *Arthrobacter* strains involved in xenobiotics degradation

Strains	Substrates		Genome availability (GenBank acc. no.)	References
	Substrates	Substrate type		
Arthrobacter sp. IF1	4-Fluorophenol	Benzenoids and their derivatives	-	Ferreira et al., 2009
Arthrobacter sp. JS443	*p*-Nitrophenol		-	Perry and Zylstra, 2007
Arthrobacter chlorophenolicus A6	4-Chlorophenol		+ (NC_011886)	Nordin et al., 2005
Arthrobacter sp. W1	Phenols		+JWMD01000000	Wang et al., 2009
Arthrobacter sp. SPG	2-Nitrobenzoate		-	Arora and Sharma, 2015
Arthrobacter sp. PJ3	Biphenyl		-	Yang et al., 2007
Arthrobacter phenanthrenivorans Sphe3	Phenanthrene		+ (NC_015145)	Vandera et al., 2015
Arthrobacter oxydans B4	Benzo[α]pyrene		-	Peng et al., 2012
Arthrobacter aurescens M2012083	Nicotine	Heterocyclic compounds	+ (AKKK00000000)	Yao et al., 2015
Arthrobacter aurescens TC1	*s*-Triazine ring compounds		+ (NC_008711)	Strong et al., 2002
Arthrobacter sp. TES6	Atrazine		-	Sebaï et al., 2011
Arthrobacter sp. SPG23	Diesel	Hydrocarbons	+ JYCN01000000	Romero et al., 2017
Arthrobacter sp. DSM312	Pentane		-	Ionata et al., 2005

Table S2 | Bacterial strains, plasmids and primers

Items	Relevant characteristics	References
**Strains**		
Arthrobacter sp. YC-RL1	CGMCC NO.10611	Ren et al, 2016
Escherichia coli DH5α	F^-, φ80, *lacZ*ΔM15, Δ(*lacZYA-argF*) U169 *endA1*, *recA1*, *hsdR17* (rk-, mk+) *supE44*, λ-, thi-1, *gyr*A96, *rel*A1, *pho*A	Promega
Escherichia coli BL21 (DE3)	F^-, *omp*T, *hsd*SB(rB^- mB^-), *gal*, *dcm* (DE3)	Promega
**Plasmids**		
pMD19T	Cloning of target gene	TaKaRa
pET32a	Expression of target gene	Novagen
**Primers**		
bphC-F	GCGC<u>GGATCC</u>ATGACTCACATCCGTGGACTT	*BamH I**
bphC-R	GCGC<u>CTCGAG</u>GTTTGCCGCGGCGAC	*Xho I**

*: The designed restriction site (the underline base pairs) in each primer.

Table S3 The detail information of chemicals involved in this study

Chemicals	CAS No.	Provider	Purity
p-Nitrophenol	100-02-7	Sinopharm Chemical Reagent Beijing Co., Ltd (Beijing, China)	Analytical grade（99.2%）
Naphthalene	91-20-3	Shanghai Aladdin Bio-Chem Technology Co., Ltd (Shanghai, China)	Analytical grade（>97.0%）
Phenanthrene	85-01-8	Shanghai Aladdin Bio-Chem Technology Co., Ltd (Shanghai, China)	Analytical grade（>97.0%）
Biphenyl	92-52-4	J&K Scientific Ltd (Beijing, China)	Analytical grade（99.5%）
Bisphenol A	80-05-7	J&K Scientific Ltd (Beijing, China)	Analytical grade（99.0%）
p-Xylene	106-42-3	Fisher Scientific	HPLC grade（99.9%）
Methanol	67-56-1	Fisher Scientific	HPLC grade（99.9%）
Acetonitrile	75-05-8	Fisher Scientific	HPLC grade（99.9%）
Salicylic acid	69-72-7	Sinopharm Chemical Reagent Beijing Co., Ltd (Beijing, China)	Analytical grade（99.3%）
Gentisic acid	490-79-9	J&K Scientific Ltd (Beijing, China)	Analytical grade（99.0%）
Benzoate	65-85-0	Sinopharm Chemical Reagent Beijing Co., Ltd (Beijing, China)	Analytical grade（99.0%）
Protocatechuate	95-50-3	J&K Scientific Ltd (Beijing, China)	Analytical grade（99.0%）
DEHP*	75-09-2	Sinopharm Chemical Reagent Beijing Co., Ltd (Beijing, China)	Analytical grade（99.0%）
Dichloromethane	141-78-6	Sinopharm Chemical Reagent Beijing Co., Ltd (Beijing, China)	Analytical grade（99.5%）

DEHP*: di 2-ethyl hexyl phthalate

Table S4 | Genomic features of the six subject *Arthrobacter* strains

Genomic features	*Arthrobacter* strain					
	YC-RL1	A6	FB24	Sphe3	TC1	RE117
Genome size (bp)	4,018,639	4,980,870	5,070,478	4,535,320	5,226,648	3,918,192
G+C content (mol %)	64.0	66.0	65.4	65.4	62.4	59.3
Accession number	NZ_CP013297.1	NC_011886.1	NC_008541.1	NC_015145.1	NC_008712.1	NC_014550.1
Protein coding genes	3,720	4,590	4,523	4,131	4,523	3,436
No. of genes with function prediction	3,579	3,095	3,256	2,922	3,256	2,378
No. of tRNAs	67	88	51	50	51	64
No. of rRNAs	19	15	15	12	15	18

Table S5 | Ring-cleavage related dioxygenases identified in strain YC-RL1

Location	Accession number	Gene annotation
Chromosome	WP_047118329	Catechol 2,3-dioxygenase
Chromosome	WP_047120477	2,3-dihydroxybiphenyl 1,2-dioxygenase
Chromosome	WP_047118329	Tryptophan 2,3-dioxygenase
Chromosome	WP_047118013	Protocatechuate 3,4-dioxygenase beta chain
Chromosome	WP_047118014	Protocatechuate 3,4-dioxygenase alpha chain
Plasmid02	WP_060617062	Hydroxyquinol 1,2-dioxygenase
Chromosome	WP_047120102	4-hydroxyphenylpyruvate dioxygenase
Chromosome	WP_047120099	3,4-dioxygenase subunit beta

Dioxygenases involved in the ring-cleavage were marked with gray background.

(I) Metabolic intermediates of BP at 24 h

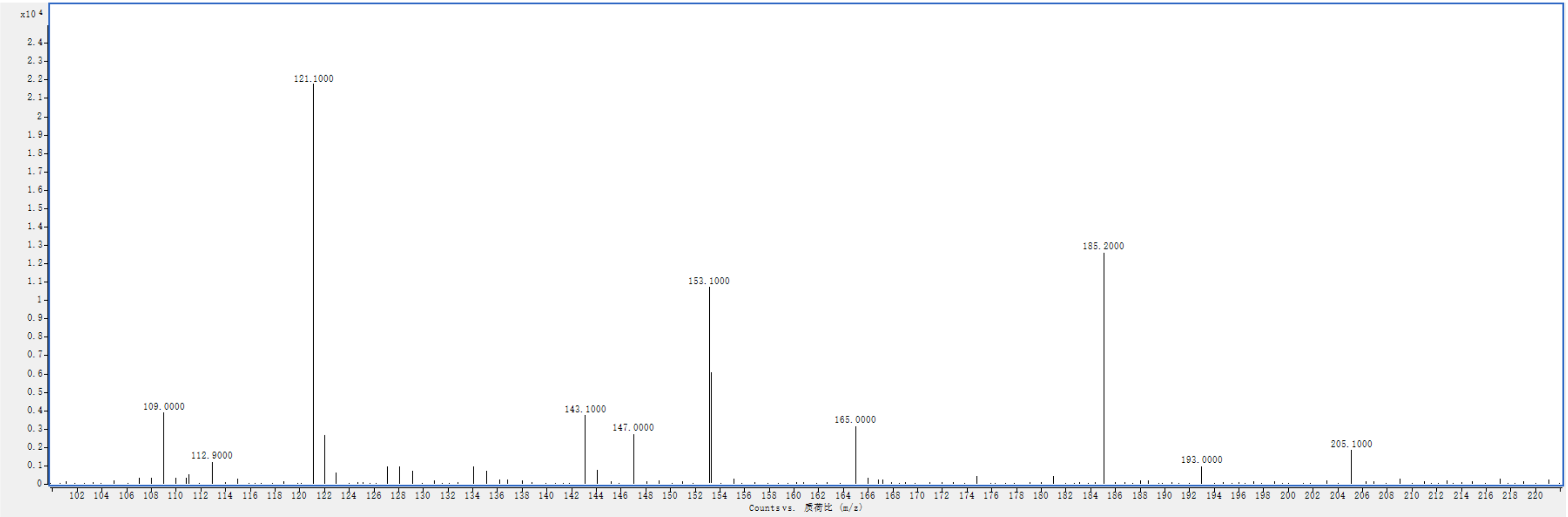

(II) Metabolic intermediates of Bisphenol A at 36 h

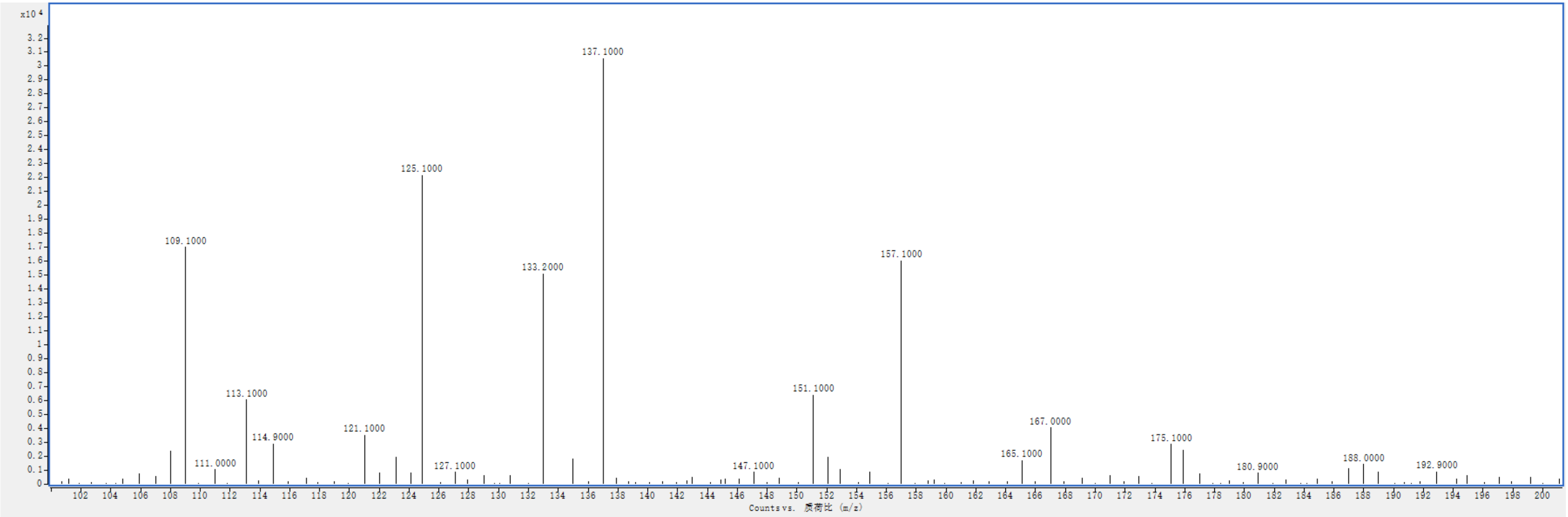

(III) Metabolic intermediates of naphthalene at 36 h

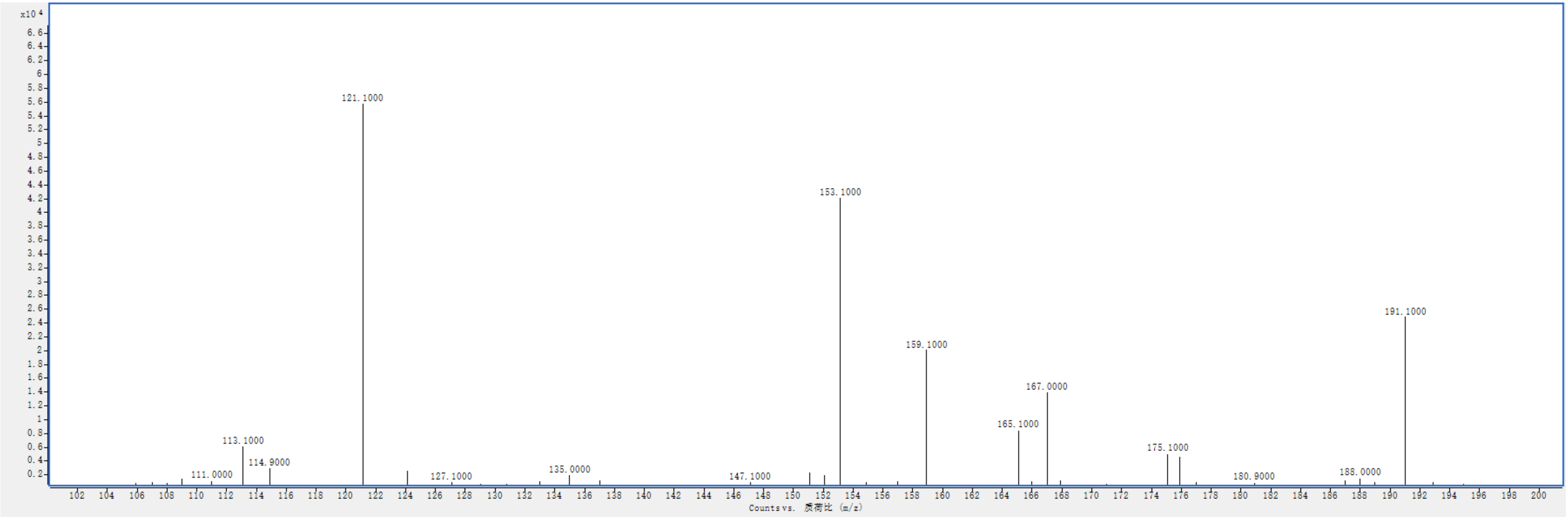

(IV) Metabolic intermediates of *p*-nitrophenol at 24h

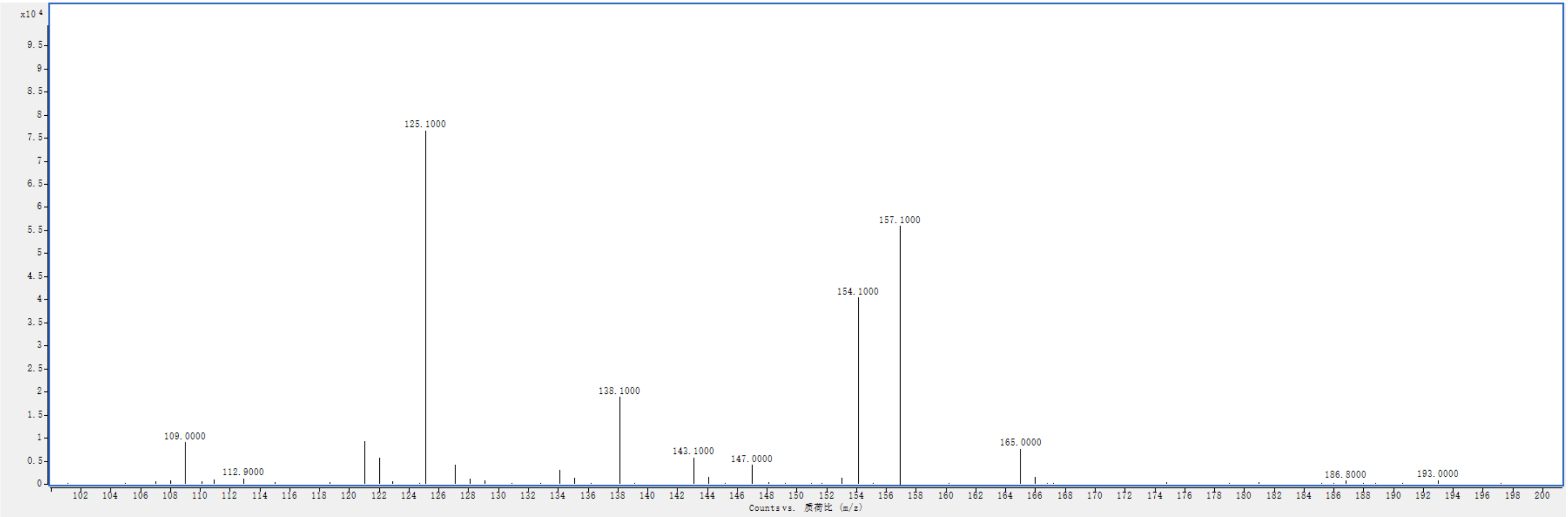

(V)HPLC analysis of benzoate, protocatechuate, salicylaldehyde and gentisic acid (standard chemicals)

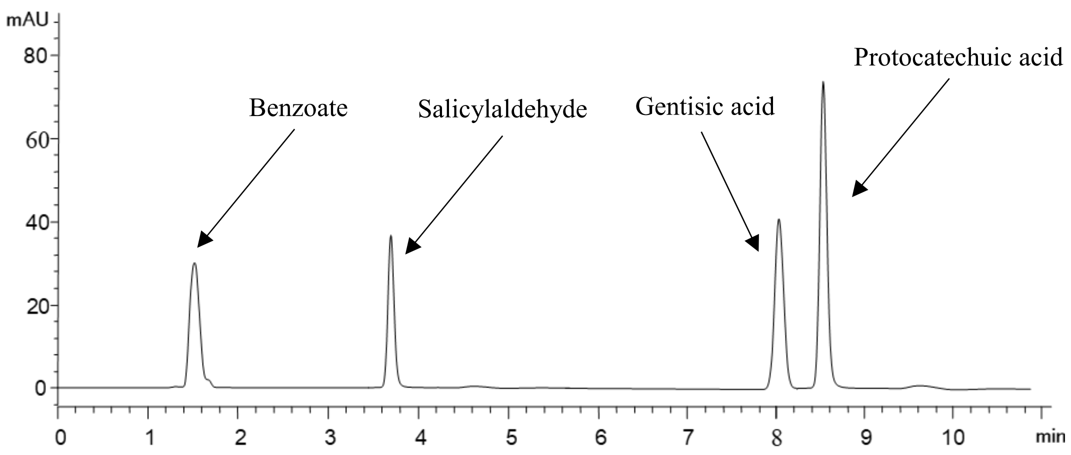

(VI) HPLC analysis of biphenyl metabolic intermediates

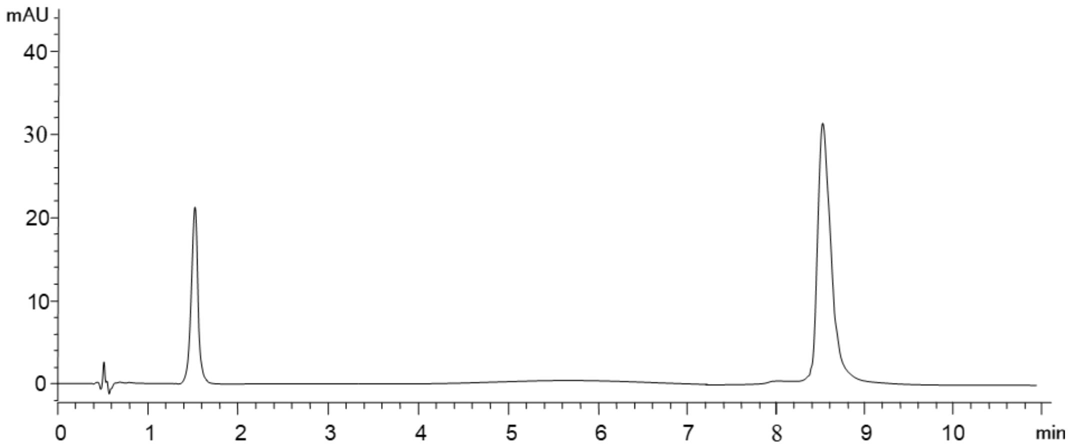

(VII) HPLC analysis of naphthalene metabolic intermediates

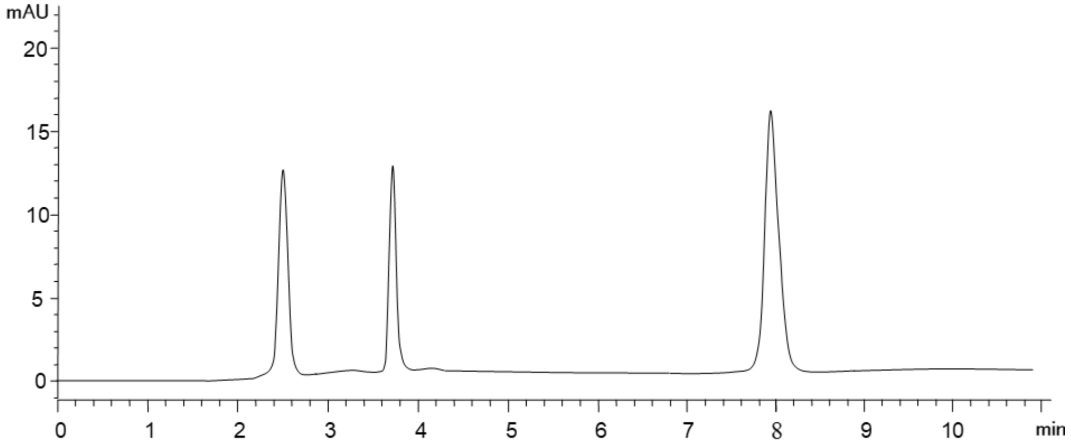

FIGURE S1 | Detection of metabolic intermediates by HPLC and HPLC-MS

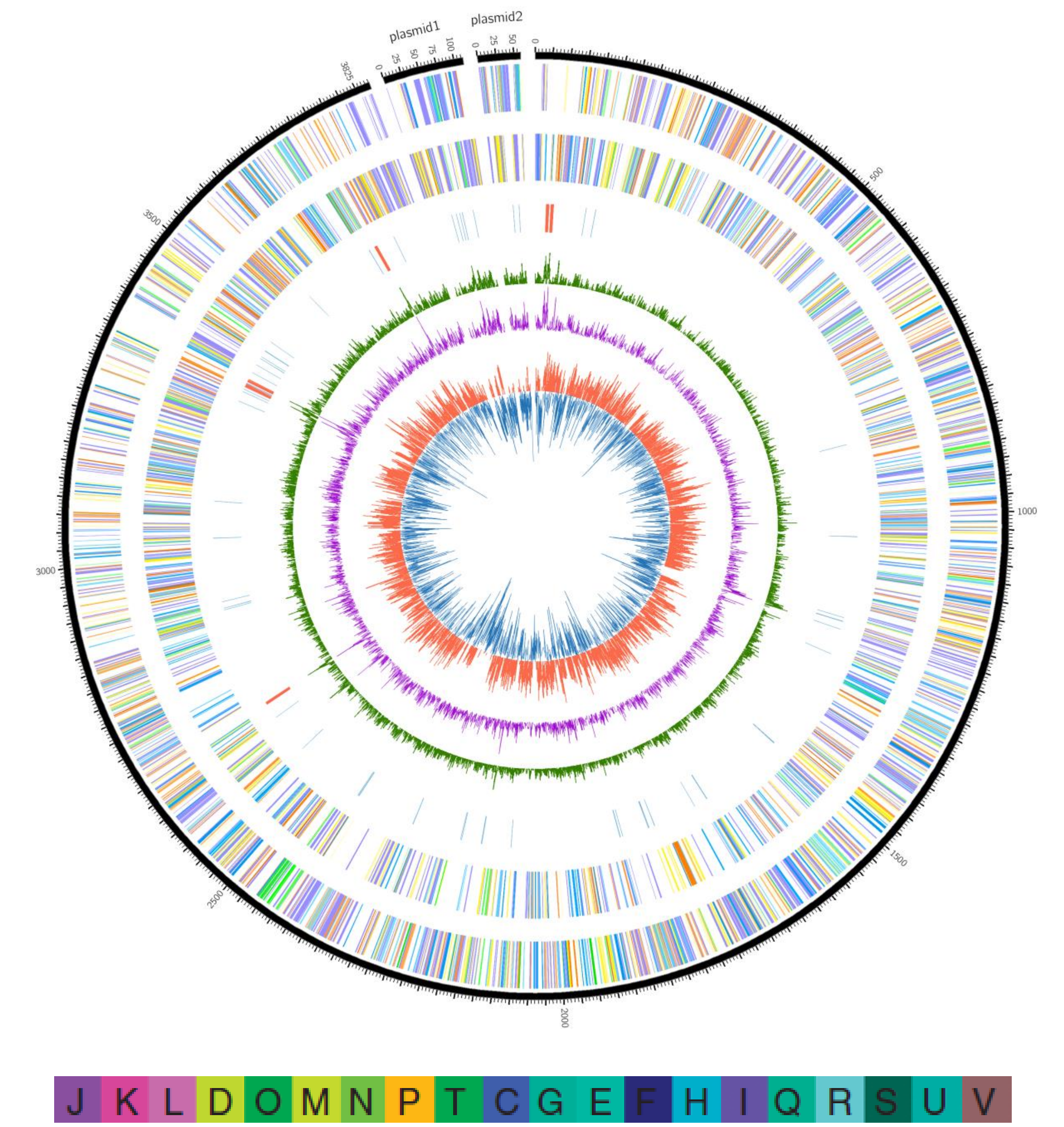

FIGURE S2 | Circular representations of strain YC-RL1 chromosome and plasmids displaying relevant genome features. From the outer to the inner concentric circle: circle 1 (outermost circle) represents the scale; circles 2 and 3, the predicted protein-coding sequences (CDS) on the forward (outer wheel) and the reverse (inner wheel) strands colored according to the assigned COG classes; circle 4 includes tRNA (blue) and rRNA (red) positions; circles 5 and 6 represent the GC skew (-) and skew (+) respectively; circles 7 and 8, G+C content showing deviations from the average (red mean higher than the average, blue means lower than the average). The bar of color indicates the COG function groups: C, energy production and conversion; D, cell cycle control, mitosis and meiosis; E, amino acid transport and metabolism; F, nucleotide transport and metabolism; G, carbohydrate transport and metabolism; H, coenzyme transport and metabolism; I, lipid transport and metabolism; J, translation; K, transcription; L, replication, recombination and repair; M, cell wall/membrane biogenesis; N, cell motility; O, post-translational modification, protein turnover, chaperones; P, inorganic ion transport and metabolism; Q, secondary metabolites biosynthesis, transport and catabolism; R, general function prediction only; S, function unknown; T, signal transduction mechanisms; U, intracellular trafficking and secretion; V, defense mechanisms.

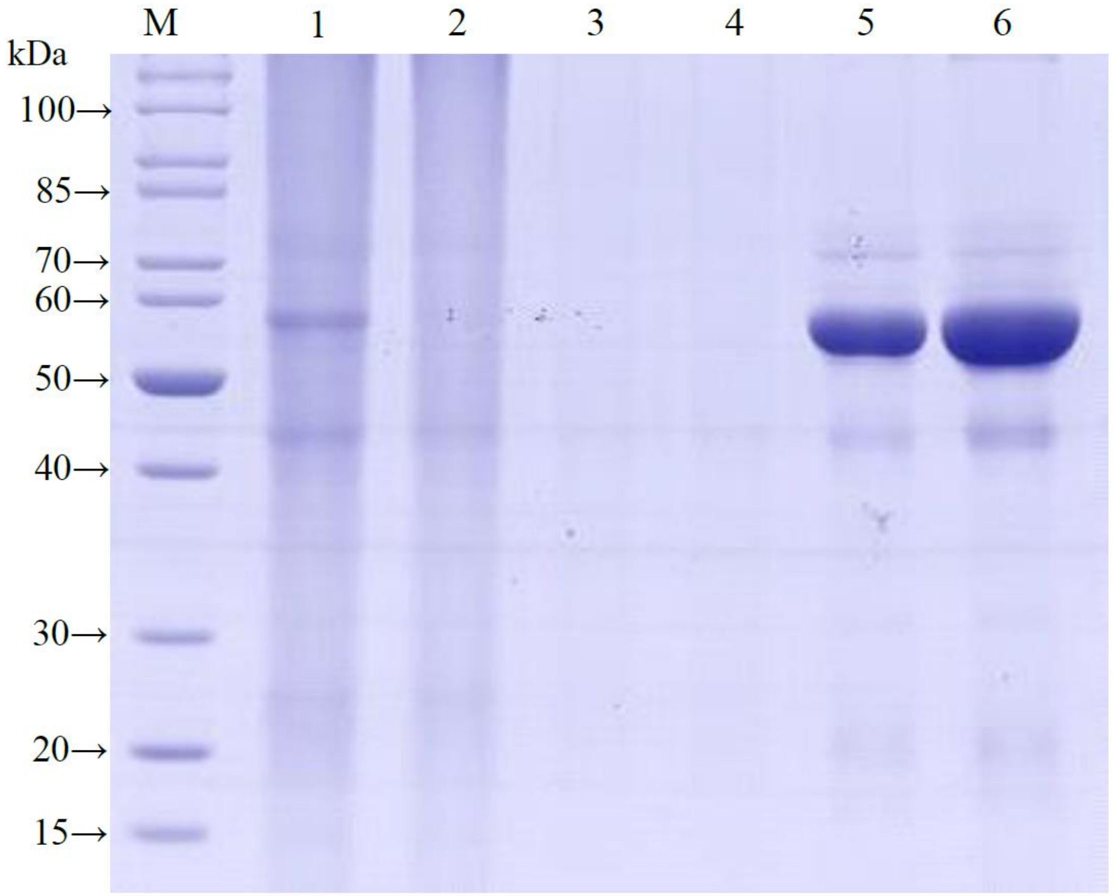

FIGURE S3| SDS-PAGE analysis of the purified BphC

(M = Protein maker, 1 = Induced E. coli BL21 cells with recombinant plasmid, 2 = Induced E. coli BL21 cells without recombinant plasmid; 3 = Purification of induced E. coli BL21 cells without recombinant plasmid, 4 = Purification of induced E. coli BL21 cells with pET32a(+) plasmid only, 5 and 6 = Purified BphC.)

References

Arora, P. K., and Sharma, A. (2015). New metabolic pathway for degradation of 2-nitrobenzoate by *Arthrobacter* sp. SPG. *Front. Microbiol.* 6:551. doi:10.3389/fmicb.2015.00551

Ferreira, M. I. M., Iida, T., Hasan, S. A., Nakamura, K., Fraaije, M. W., Janssen, D. B., and Kudo, T. (2009). Analysis of two gene clusters involved in the degradation of 4-fluorophenol by *Arthrobacter* sp. strain IF1. *Appl. Environ. Microbiol.* 75, 7767-7773. doi: 10.1128/AEM.00171-09

Ionata, E., Blasio, P., and Cara, F. (2005). Microbiological degradation of pentane by immobilized cells of *Arthrobacter* sp. *Biodegradation* 16, 1-9. doi: 10.1007/s10531-004-0473-x

Nordin, K., Unell, M., and Jansson, J. K. (2005). Novel 4-chlorophenol degradation gene cluster and degradation route via hydroxyquinol in *Arthrobacter chlorophenolicus* A6. *Appl. Environ. Microbiol.* 71, 6538-6544. doi: 10.1128/AEM.71.11.6538-6544.2005

Peng. H., Yin. H., Deng. J., Ye. J. S., Chen. S. N., He. B. Y., and Zhang. N. (2012). Biodegradation of benzo[α]pyrene by *Arthrobacter oxydans* B4. *Pedosphere* 22, 554-561. doi: 10.1016/S1002-0160(12)60040-X

Perry, L. L., and Zylstra, G. J. (2007). Cloning of a gene cluster involved in the catabolism of *p*-nitrophenol by *Arthrobacter* sp. strain JS443 and characterization of the *p*-nitrophenol monooxygenase. *J. Bacteriol.* 189, 7563-7572. doi: 10.1128/JB.01849-06

Ren, L., Jia, Y., Ruth, N., Zhao, B., and Yan, Y. (2016). Complete genome sequence of an aromatic compound degrader *Arthrobacter* sp. YC-RL1. *J. Biotechnol.* 219, 34-35. doi: 10.1016/j.jbiotec.2015.12.008

Romero, R. M., Gkorezis, P., Kidd, S. P., Hamme, V. J., Weyens, N. Monterroso, C., and Vangronsveld J. (2017). Characterization and degradation potential of diesel-degrading bacterial strains for application in bioremediation. *Int. J. Phytoremediation*. 19, 955-963. doi: 10.1080/15226514.2017.1337065

Sebaï, E. T., Lamrani, D. M., Changey, F., Rouard, N., and Laurent M. F. (2011). Evidence of atrazine mineralization in a soil from the Nile Delta: Isolation of *Arthrobacter* sp. TES6, an atrazine-degrading strain. *Int. Biodeterior. Biodegradation*. 65, 1249-1255. doi: 10.1016/j.ibiod.2011.05.011

Strong, L. C., Rosendahl, C., Johnson, G., Sadowsky, M. J., and Wackett, L. P. (2002). *Arthrobacter aurescens* TC1 metabolizes diverse *s*-triazine ring compounds. *Appl. Environ. Microbiol.* 68, 5973-5980. doi: 10.1128/AEM.68.12.5973-5980.2002

Vandera, E., Samiotaki, M., Parapouli, M., Panayotou, G., and Koukkou, I. A. (2015). Comparative proteomic analysis of *Arthrobacter phenanthrenivorans* Sphe3 on phenanthrene, phthalate and glucose. *J. Proteomics* 113, 73-89. doi: 10.1016/j.jpr ot.2014.08.018

Wang, P., Qu, Y., and Zhou, J. (2009). Biodegradation of mixed phenolic compounds under high salt conditions and salinity fluctuations by *Arthrobacter* sp. W1. *Appl. Biochem. Biotechnol.* 159, 623-633. doi: 10.1007/s12010-008-8494-7

Yang, M., Ma, P., Li, W., Liu, J., Li, L., Zhu, X., and Wang, X. (2007). 2, 3-Dihydroxybiphenyl dioxygenase gene was first discovered in *Arthrobacter* sp. strain PJ3. *Chinese Science Bulletin* 52, 1205-1211. doi: 10.1007/s11434-007-0191-3

Yao, Y., Tang, H., Su, F., and Xu, P. (2015). Comparative genome analysis reveals the molecular basis of nicotine degradation and survival capacities of *Arthrobacter*. *Sci. Rep.* 5, 8642. doi: 10.1038/srep08642
